# Proteome Analysis of *Corynebacterium diphtheriae*–Macrophage Interaction

**DOI:** 10.1002/pmic.202400317

**Published:** 2025-04-10

**Authors:** Luca Musella, Jens Möller, Christopher Lischer, Julio Vera‐Gonzalez, Jörg Hofmann, Lisa Ott, Andreas Burkovski

**Affiliations:** ^1^ Microbiology Division Department of Biology Friedrich‐Alexander‐Universität Erlangen‐Nürnberg Erlangen Germany; ^2^ Laboratory of Systems Tumor Immunology Friedrich‐Alexander‐Universität Erlangen‐Nürnberg Deutsches Zentrum Immuntherapie BZKF and Universitätsklinikum Erlangen Erlangen Germany; ^3^ Biochemistry Division Department of Biology Friedrich‐Alexander‐Universität Erlangen‐Nürnberg Erlangen Germany

**Keywords:** host–pathogen interaction, mycobacteria, phagocytosis, proteomics, reactive oxygen species

## Abstract

Contact of *Corynebacterium diphtheriae* with macrophages induces adaptations on both bacterial and cellular sides. The study presented here was aiming to shed light on the simultaneous intracellular adaptation of the bacteria and changes in the proteome of the phagocytes in response to the internalization of *C. diphtheriae*. Quantitative proteome analyses were carried out at different time points of an infection assay and data were analyzed by different bioinformatic approaches. Several *C. diphtheriae* proteins, which were not observed or connected with pathogenicity before, were differentially expressed, as well as key macrophage components of the phagolysosome. Overall, bacteria responded to phagocytosis by changes in DNA repair, transcription, and cell wall synthesis proteins, while macrophages showed changes in components of the innate immune system.

## 1 Introduction

Diphtheria is a disease of the upper respiratory tract caused by toxigenic *Corynebacterium diphtheriae*. As a consequence of infection, a pseudo‐membrane is formed on tonsils and throat from aggregated damaged host cells and bacteria, leading to breathing difficulties and putative death by suffocation [1, 2, 3, 4]. The main virulence factor of *C. diphtheriae* is the highly potent diphtheria exotoxin, which is acquired by infection of strains with lysogenic corynebacteriophages bearing the tox gene and expressed in response to iron limitation [5, 6]. Diphtheria toxin catalyzes the ADP‐ribosylation of peptide elongation factor EF‐2, leading to a block of proteins translation by impeding the transfer of tRNA‐loaded amino acids to the nascent peptide chain [7, 8]. Due to the impairment of protein synthesis, one molecule of cytosol‐imported exotoxin is sufficient to kill a cell [9].

Phylogenomically, *C. diphtheriae* is highly diverse with global cocirculation of multiple toxigenic and nontoxigenic lineages [10]. Interestingly, nontoxigenic strains without the capacity to produce diphtheria toxin are increasingly observed in the context of systemic infections such as endocarditis, osteomyelitis, sepsis, and septic arthritis [11, 12, 13, 14]. Within the human body, these invasive strains are prone to destruction by phagocytes. However, when compared to other important pathogens like *Mycobacterium tuberculosis*, relatively little is known about the exact interaction between human macrophages and nontoxigenic *C. diphtheriae* strains. Dos Santos et al. provided evidence of nonopsonic internalization of *C. diphtheriae* by human macrophages. Moreover, it was shown that the tox^−^ strain ATCC 27010, although possessing less effective adhesion and invasion properties, was able to survive up to 24 h in U‐937 macrophages and to induce the death of host cells, by both apoptosis and necrosis [15]. These findings were more recently confirmed by Weerasekera et al. using THP‐1 cells and *C. diphtheriae* strain HC04 [16]. A study using nontoxigenic strains highlighted DIP0733, a multifunctional virulence factor of *C. diphtheriae* crucial for interaction with epithelial cells and pathogenicity in invertebrate animal model systems, as a relevant protein in the process of internalization of *C. diphtheriae* in THP‐1 macrophages [17, 18], while different nontoxigenic strains may also possess different internalization rates, thus suggesting an underlying diversity in their repertoire of invasion factors and consequent pathogenicity [19]. On the host side, C‐type lectin receptor Mincle has been recently identified to recognize *C. diphtheriae* by binding a yet unidentified glycolipid component of the cell wall. Taken together with the evidence that TLR2 also recognizes cell wall constituents of the bacterium, these findings may provide the first evidence of a coexistence of pro‐ and antiinflammatory signals in host–pathogen interaction, thus mitigating the bactericidal activity of macrophages when infected by *C. diphtheriae*. The importance of TLR signaling is further highlighted by studies with cells deficient of the cytoplasmic adaptor Myd88, which appears to be essential for the production of high levels of the cytokines G‐CSF and IL‐6, as well as for the internalization of tox^−^ strains such as ISS3319. In particular, *C. diphtheriae* ISS3319 is known to induce the NF‐kB signaling pathway upon internalization in NF‐kB reporter cells and macrophages [20]. Finally, confocal microscopy experiments showed that the colocalization of *C. diphtheriae* strains with acidic compartments was delayed, compared to nonpathogenic *C. glutamicum*, thus suggesting that the bacterium was able to impair phagosome‐lysosome fusion. Interestingly, in contrast to the mycobacterial cord factor, corynebacterial mycolic acids seem to be not involved in this process [21].

**Summary**

• In the frame of this study, the simultaneous adaptation of *C. diphtheriae* and phagocytes in response to pathogen internalization was characterized for the first time on the protein translation level.

To gain more insights into the interaction between nontoxigenic *C. diphtheriae* strain ISS3319 and human THP‐1 macrophage cells during infection, a proteomics study was carried out. In contrast to previous studies, which focused on the effect of distinct virulence factors on the infection process or on the proteomics of *C. diphtheriae* alone [22, 23, 24, 25], the approach presented here was aiming to shed light on the simultaneous intracellular adaptation of the bacteria and changes in the proteome of the phagocytes in response to internalization of *C. diphtheriae*.

## 2 Materials and Methods

### 2.1 Experimental Design

**FIGURE 1:**
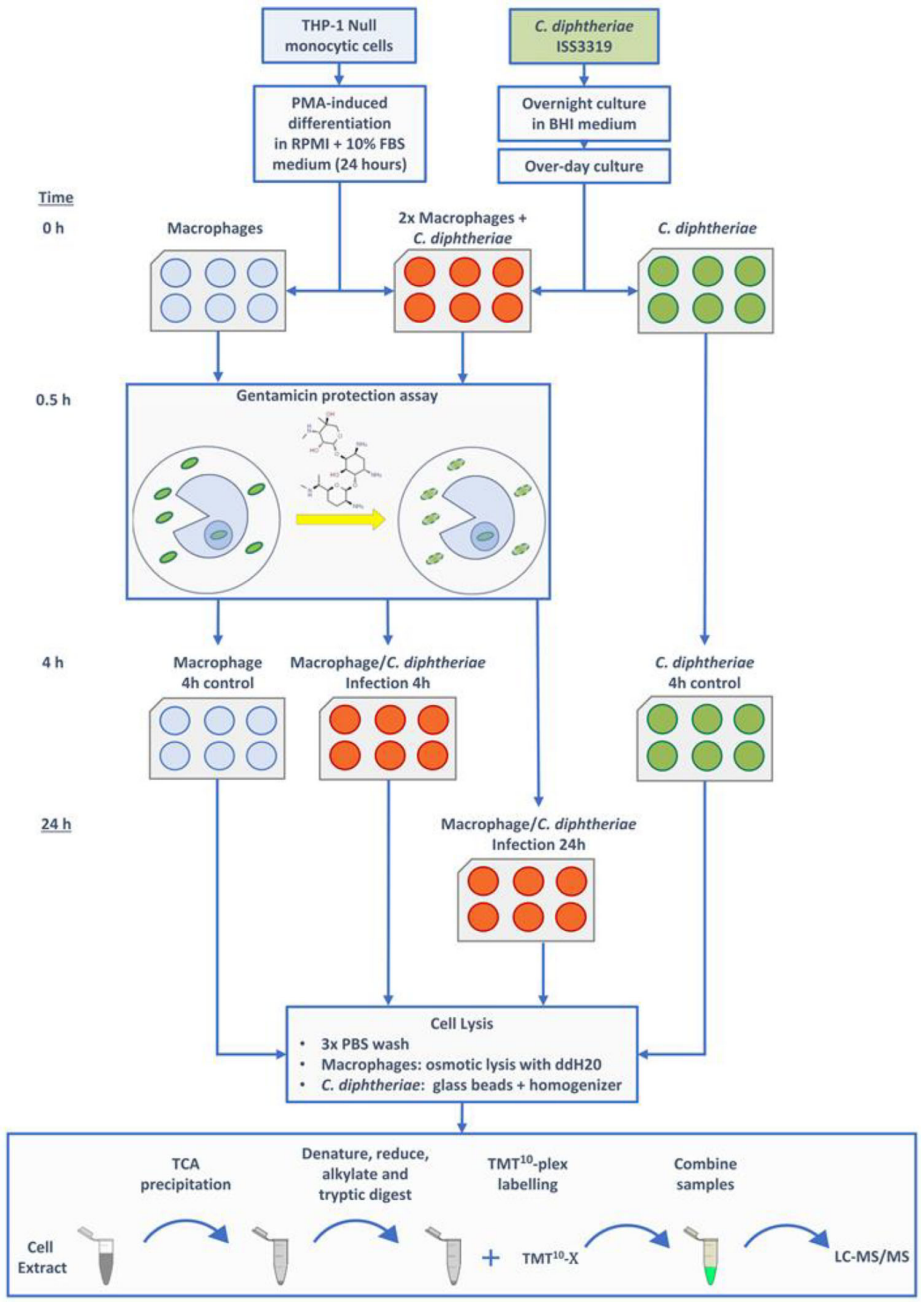
Experimental setup.

An overview of the experimental design is provided in Figure 1. For each condition (macrophage 4 h control, *C. diphtheriae* 4 h control, macrophage and *C. diphtheriae* 4 h infection, and macrophage and *C. diphtheriae* 24 h infection), three biological replicates were carried out.

THP1‐Null monocytic cells (Invivogen, France) were cultured in Rosewell Park Memorial Institute (RPMI medium, ThermoFisher, Germany) supplemented with 10% fetal bovine serum (FBS, ThermoFisher). Differentiation of THP1‐Null cells into macrophage was induced by phorbol 12‐myristate 13‐acetate (PMA). *C. diphtheriae* ISS3319 was cultured in Brain Heart Infusion (BHI, Oxoid, Germany) medium, and inoculums for the infection were prepared using bacterial cultures in the exponential phase.

For infection assays, a multiplicity of infection (MOI) of 200 was used (i.e., 1.6 × 10^6^ macrophages per well were differentiated and 3.2 × 10^8^ bacteria were inoculated subsequently), which is higher than the typically used MOIs of 1–100, to improve detection of proteins from phagocytosed bacteria. The plates were centrifuged for 5 min at 350 x *g* to synchronize infection and incubated for 30 min (37°C, 5% CO_2_, 90% humidity) to allow phagocytosis of bacteria. Subsequently, the supernatant containing nonengulfed bacteria was aspirated, cells were washed once with PBS and remaining extracellular bacteria were killed by the addition of 100 µg mL^−1^ gentamicin. After 2 h, cells were either lysed and intracellular bacteria were recovered or further incubated with a medium containing 10 µg mL^−1^ gentamicin for 4 and 24 h.

### 2.2 Cell Lysis and Protein Sample Preparation

To separate human and *C. diphtheriae* proteins, macrophages were lysed in a first step osmotically via ddH_2_O incubation. After centrifugation, the supernatant containing the macrophage proteins was collected and the remaining intact corynebacteria in the pellet were subsequently disrupted by homogenization using glass beads [26, 27]. Total protein extracts were prepared as described [28]. For each condition and specimen, 40 µg of material was collected for further processing.

TMT‐10 (ThermoFisher) was used to uniquely tag two technical replicates for each control condition and three technical replicates for each infection condition using the protocol of the supplier. Briefly, after equilibrating the TMT label reagents at room temperature, the labeling solution was added to 100 µL sample aliquots and the solution was incubated for 1 h at room temperature. The reaction was quenched by adding 5% hydroxylamine to the sample and incubating for 15 min, then the TMT‐labeled samples were combined and vacuum‐concentrated until dryness.

### 2.3 Liquid Chromatography and Mass Spectrometry

A TMT10‐plex label can react with a free amine group of a peptide, resulting in an amide bond. Being isobaric, the mass tags do not cause differences between samples when analytes reach the quadrupole mass analyzer; conversely, secondary ion fragmentation followed by Orbitrap analysis reveals the sample‐specific mass reporter, as its bond with the mass normalizer is cleaved. This allowed to analyze conditions from the same biological replicate in one LC‐MS/MS run, which can mitigate the technical variability associated to performing consecutive runs of tandem mass spectrometry. Conversely, secondary ion fragmentation (MS3) followed by Orbitrap analysis reveals the sample‐specific TMT‐mass reporter, as its bond with the mass normalizer is cleaved. MS3 tandem mass spectrometry was carried out similarly to what previously described [29]. Ten microgram peptide aliquots were loaded onto a nanoflow Ultimate 3000 HPLC (Dionex, Sunnyvale, CA, USA). For the separation of peptides, an EASY‐Spray column (C18, 2 µm particle size, 50 cm × 75 µm) was used (flow rate: 200 nL min^−1^; increasing acetonitrile concentrations over 120 min). The total method duration (including equilibration and column wash) was 160 min. Detection of mass spectra was carried out using an Orbitrap Fusion mass spectrometer (Thermo Fisher Scientific, Germany) with the following settings: Spray voltage 2000 V, transfer tube temperature 275°C, scan range for the MS2 detection in the Orbitrap 300–2000 (*m*/*z*), 50 ms maximum injection time, automatic gain control (AGC) target of 400,000 ions, and Orbitrap resolution of 120,000. The most intense ions were selected for collision‐induced dissociation (CID) with a collision energy of 35%. For ion trap detection, a maximum injection time of 250 ms and an AGC target of 100 ions were set [30]. Secondary ion fragmentation combined with Orbitrap analysis (MS3) reveals the sample‐specific TMT‐mass reporter, as its bond with the mass normalizer is cleaved. MS3 scan parameters for the TMT‐label were as follows: Higher‐energy collisional dissociation (HCD) (collision energy 65%), Orbitrap resolution 60,000, scan range 120–500, AGC target 1.2e5 ions, and max injection time 120 ms. The resulting raw data files have been deposited at MassIVE (ProteomeExchange Id. PXD061792, ftp://massive.ucsd.edu/v09/MSV000097308/).

### 2.4 Computational Methods

#### 2.4.1 Detection of Peptides From Mass Spectra and Peptide‐to‐Protein Assignment

The resulting raw data were analyzed using the Proteome Discoverer 1.4 program package (Thermo Fisher Scientific). The theoretical masses of peptides were generated with a maximum of two missed cleavages. Carbamidomethyl modification on cysteine was set as a fixed modification, oxidation of methionine as a dynamic modification. For fragment mass measurements, the mass tolerance parameters for survey scans used to compare spectra of product ions were set to 10 ppm and 0.6 Da. For peptide sequencing, in silico tryptic digestion of known protein sequences belonging to two annotated UniProt [31] proteomes was used (*C. diphtheriae* ATCC 700971/NCTC 13129/Biotype gravis database, Proteome ID UP000002198; *Homo sapiens*, Proteome ID UP000005640). The available Sequest HT algorithm was used for peptide search. Additionally, a target‐decoy search strategy was applied [32]. The program was then allowed to freely choose the best match. It was possible to estimate the proportion of false positives from the number of matched decoy peptides with a score higher than a given threshold. A similar argument addressed the case of false negatives, which can be controlled by adjusting the sensitivity of the pipeline. A false discovery rate (FDR) of 1% for peptide identification was chosen. Concerning the assignment of peptides to proteins, it was chosen to utilize unique peptides which are not present in other sequences, plus a restricted set of shared ones, called by the software “razor peptides”. Then, by simple summation, the protein abundance was computed. The final output is an Excel file containing general information for each identified protein, such as the UniProt database identifier, the molecular weight, the number of detected peptides and corresponding TMT‐10 tag, and calculated abundances for control and infection groups. Separate output files were generated for the corresponding reference proteomes. The data is openly available at https://zenodo.org/doi/10.5281/zenodo.12706139 and MassIVE (ProteomeExchange Id. PXD061792, ftp://massive.ucsd.edu/v09/MSV000097308/).

#### 2.4.2 Statistical Analysis of Proteomics‐Derived Protein Abundances

To perform differential protein abundance analysis from Proteome Discoverer outputs, filtering and normalization were performed for each set of specimen‐specific samples, that is, three conditions for each biological replicate, for a total of nine samples. Zero‐replacement imputation was employed [33], then we excluded from subsequent steps proteins whose abundance was different from zero in less than four samples, as this guarantees that at least one non‐zero abundance in at least two conditions is presented in the selected proteins. After filtering, as LC‐MS/MS data tend to be lognormal distributed [34], data were transformed by applying the base‐two‐logarithm to the sum between the protein abundance and a positive constant (0.1), in order to avoid negative infinite values in the case of abundance values of zero. Next, total abundance and quantile normalization across samples and conditions were performed to further control differences between samples [35]. Then, the LIMMA‐trend framework, without robustified parameter estimation, was employed to estimate fold‐changes and therefore differential protein abundances between control and infection groups for each specimen [36]. Briefly, a linear model that accounts for condition and batch was constructed, as the three biological replicates were processed in two distinct LC‐MS/MS runs: this allowed to correct for batch effects when comparing biological conditions. Additionally, a trend curve that explains protein‐wise variance in abundance as a function of the average protein‐wise log‐abundance value was fitted and employed as prior variance distribution, which is used for the moderated *t* test statistics to assess differential protein abundance.

Proteins excluded from the differential protein abundance analysis and that only showed non‐zero abundance in at least two biological replicates from a single condition were classified as de novo synthesized.

To conduct gene set enrichment analysis (GSEA), the R library *Cluster‐Profiler* [37] and its implementation of *fgsea* [38] were utilized to perform the statistics and generate the plots. As gene sets, Reactome pathways and KEGG BRITE hierarchies of sizes ranging from 3 to 100 were respectively used for macrophages and *C. diphtheriae*. The former was obtained by querying the Reactome Graph Database using Neo4j, the latter via the R library *KEGGREST* [39, 40, 41].

An FDR of 5% was chosen for all statistical analyses.

#### 2.4.3 Data Mining and Functional Protein Characterization

The UniProt database was used to access annotated PFAM and InterPro domains Gene Ontology (GO) terms for the differentially abundant proteins. To facilitate the interpretation of mostly sparse and highly nonredundant “Molecular Function” GOs, a search for a minimal, representative set of GOs was conducted. Briefly, as GOs are hierarchically organized, with child nodes representing increasingly narrower concepts than their respective parent nodes, it is always possible to find a last common ancestor (LCA) of a given collection of GOs. Then, the representative ancestor set of GOs was constructed by iterative expansions, starting from the LCA, one neighborhood order at a time, until at least *k* GOs were found, such that each original GO is represented by an ancestor GO. The parameter *k* was set to 4, that is, the minimal, representative ancestor set must be of size 4 or greater. The analysis was conducted using the UniProt [UniProt Consortium] annotations available by May 2024 and the GO Database available by November 2023 [42].

To further characterize *C. diphtheriae* differentially abundant proteins, UniProt‐annotated PFAM and InterPro domains were also retrieved. Predicted protein clans were then assigned using PFAM's available domain‐to‐clan associations, as available by March 2025 [43]. Further, InterPro‐domain‐derived GOs were assigned based on the latest database iteration [44]. Furthermore, a BLASTP search was performed for each differentially abundant bacterial protein to find a homolog in *C. diphtheriae* bv. *mitis*. *E* Value and maximum number of returned hits were 1e‐5 and 50, respectively. Hits were further filtered by imposing 99% query coverage and 95% identity [45].

Finally, STRING's v12 [46] protein–protein interaction (PPI) network for *C. diphtheriae* NCTC13129 was used to predict functional associations between differentially abundant proteins. The functional associations were first pruned by selecting only *neighborhood*, *fusion*, *coexpression*, *experimental*, and *database* channels, then a cutoff of 0.4 was applied. Nodes were also filtered by only retaining proteins whose abundance was different from zero in at least four samples or de novo translated. The resulting connected network consisted of 1067 nodes and 20,594 edges. Next, we used *igraph* [47, 48] implementations of Dijkstra's algorithm to retrieve all weighted, all‐pairs shortest paths (APSP) between differentially abundant proteins. Network community structure was inferred using Leiden clustering [49]. After that, each community was tested for over‐representation of KEGG's BRITE hierarchies using a 2 × 2 contingency table and Fisher's exact test (5% FDR *p* value adjustment) [40]. The nodes in the APSP‐derived network constituted the test *universe*. Before statistical testing, protein sets were required to contain at least three proteins included in the APSP‐derived network and aggregated into a unique gene set if identical after removing proteins that were not in the *universe*. BRITE hierarchies that were significantly associated to a community and showing the highest proportions of overlapping proteins were used as a proxy to functionally describe the community.

The data and the data mining scripts are openly available at https://zenodo.org/doi/10.5281/zenodo.12706139 and MassIVE (ProteomeExchange Id. PXD061792, ftp://massive.ucsd.edu/v09/MSV000097308/).

## 3 Results

### 3.1 Growth and Viability of *C. diphtheriae* ISS3319 in Cell Culture Medium

*C. diphtheriae* is not only able to grow in cell culture medium but it also adapts its metabolism to different cell culture medium components. Mass spectrometric analyses and label‐free protein quantification hint at an increased bacterial pathogenicity when *C. diphtheriae* is grown in RPMI and fetal calf serum as well as an influence of the growth medium on the cell envelope [50].

**FIGURE 2:**
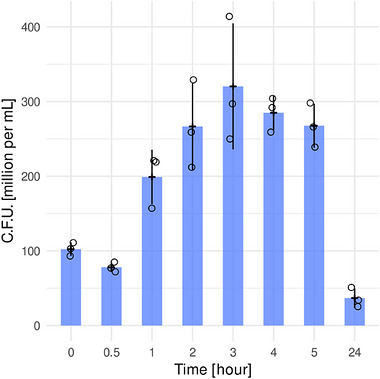
Growth and viability of *C. diphtheriae*. Strain ISS3319 was grown in Rosewell Park Memorial Institute (RPMI) medium with 10% fetal bovine serum. At each indicated time point, bacteria were plated on BHI agar plates after serial dilutions to determine colony forming units (CFU). Means and standard deviations of three biological replicates are shown.

As a basis for a proteomic characterization of *C. diphtheriae* interaction with macrophages, growth and viability of *C. diphtheriae* in RPMI and fetal calf serum was tested. When *C. diphtheriae* was grown under cell culture conditions in 6‐well plates, the optical density at 600 nm (OD_600_) increased consistently over the course of 5 h. Overnight incubation moderately reduced the OD_600_ value. When bacterial viability was tested, the number of viable cells increased as well, but reached a plateau between 3 and 4 h of incubation, indicating a progressive inactivation of the bacteria in cell culture medium at later time points (Figure 2).

### 3.2 Response of *C. diphtheriae* ISS3319 to Internalization by Macrophages

In this study, a total of 1358 unique bacterial proteins were detected (Table 1), 1136 of which were suitable for performing differential abundance analysis, out of the 2265 annotated under the reference proteome of *C. diphtheriae* NCTC13129 (UniProt ID UP000002198) and of the annotated 2257 proteins of *C. diphtheriae* strain ISS3319 [51], respectively, indicating a high coverage of the expressed proteome. Although the 4 h control and infection conditions show the same number of detected proteins, fewer proteins were detected in the 24 h infection group (Table 1). When the proteome of *C. diphtheriae* ISS3319 incubated for 4 h in cell culture medium was compared to the proteome of bacteria phagocytosed by macrophages for the same time, no statistically significant differences in protein abundance were observed. This may be explained by the fact that cell culture medium components lead to a preadaptation of *C. diphtheriae* to host cell contact [50].

**TABLE 1:** Counts of detected and undetected proteins across biological replicates and conditions.

		Detected proteins					
Cell	Condition	Undetected	One replicate	2x Overlap	3x Overlap	Detected total	Union
THP‐1 null macrophages	4 h control	141	2627	3556	4558	10,741	10,882
	4 h infection	141	2627	3556	4558	10,741	
	24 h infection	6073	1781	1281	1747	4809	
*C. diphtheriae*	4 h control	18	212	335	793	1340	1358
	4 h infection	18	212	335	793	1340	
	24 h infection	484	209	175	490	874	

**FIGURE 3:**
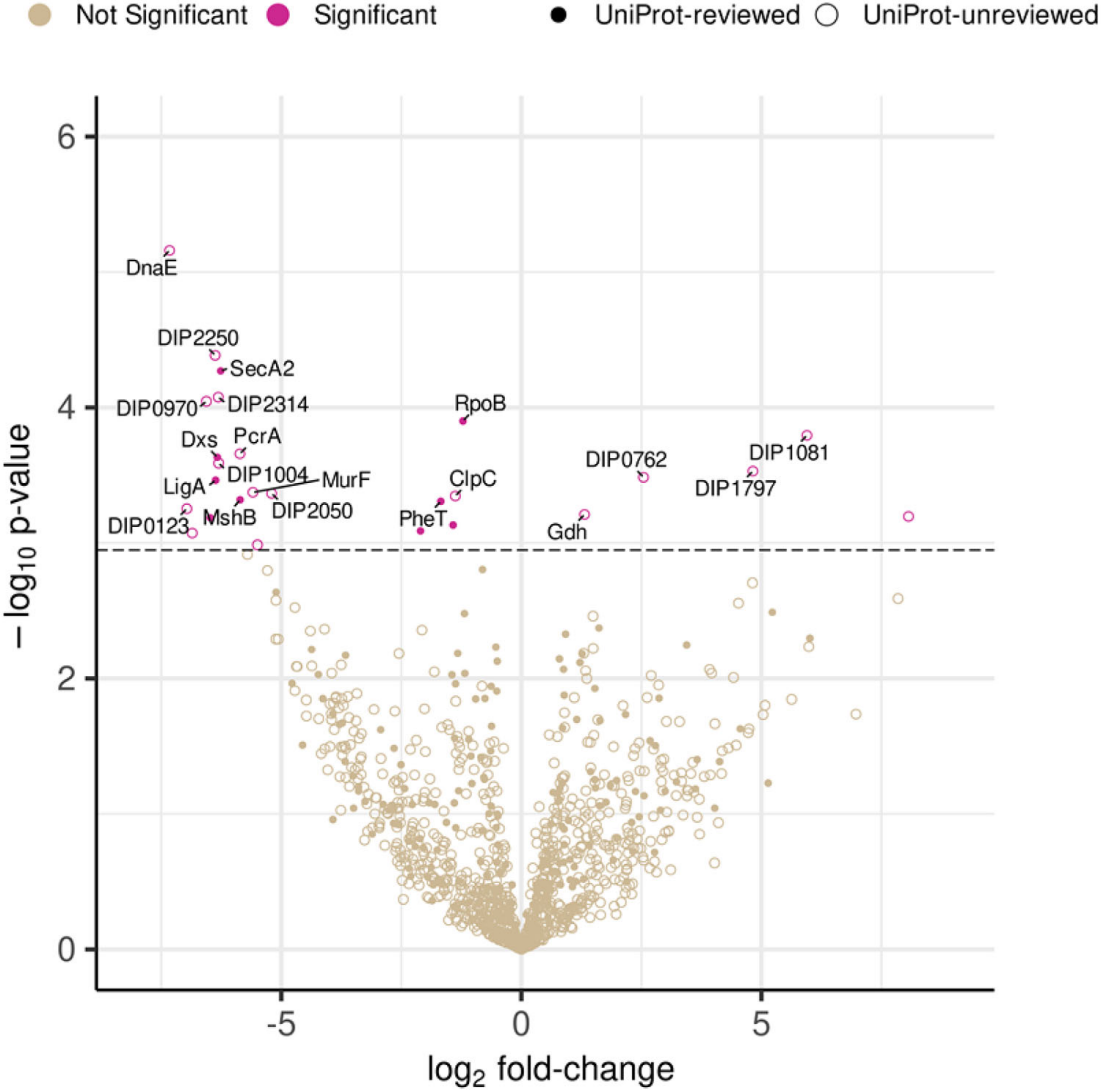
Differential protein abundance in *C. diphtheriae* between 4 and 24 h of internalization. Log‐transformed *p* values and logarithmic fold‐changes of 1134 analyzed proteins are shown. The horizontal dashed line corresponds to the log‐transformed critical *p* value corresponding to 5% false discovery rate (FDR). Twenty differentially abundant proteins with lowest *p* values are indicated.

It was reported previously that even after 24 h of internalization, *C. diphtheriae* can survive in macrophages by the prevention of phagolysosome maturation. Responsible proteins or other factors were unknown [21]. Comparison of *C. diphtheriae* internalized for 4 and 24 h revealed 5 proteins with higher abundance and 21 proteins with lower abundance out of 1136 evaluated proteins (Table 2, Figure 3).

**TABLE 2:** *C. diphtheriae* proteins with differential abundance between 4 and 24 h of internalization.

UniProt ID (protein name), original	Gene name	LFC	UniProt ID (protein name), bv. *mitis* homolog	PFAM clans	InterPro‐to‐GO
Q6NEC4 (Tryptophan synthase beta chain)	*TrpB*	8,1	A0A854NJH1 (Tryptophan synthase beta chain)	FAD/NAD(P)‐binding Rossmann fold superfamily (CL0063.25)	Tryptophan synthase activity (GO:0004834)
Q6NHQ0 (Acetyltransferase)	*DIP1081*	5,9	A0A854NHA6 (Acetyltransferase)	*N*‐Acetyltransferase like (CL0257.9)	Acyltransferase activity, transferring groups other than amino‐acyl groups (GO:0016747)
Q6NFT9 (Uncharacterized protein)	*DIP1797*	4,8	A0A854NE82 (Disulfide bond formation protein DsbA)	Thioredoxin‐like (CL0172.17)	/
Q6NIK7 (Methyltransferase type 11 domain‐containing protein)	*DIP0762*	2,5	A0A854NM12 (Methyltransferase)	FAD/NAD(P)‐binding Rossmann fold superfamily (CL0063.25)	S‐adenosylmethionine‐dependent methyltransferase activity (GO:0008757)
Q6NGH0 (Glutamate dehydrogenase)	*Gdh*	1,3	A0A854NC31 (Glutamate dehydrogenase)	FAD/NAD(P)‐binding Rossmann fold superfamily (CL0063.25), aminoacid dehydrogenase‐like, N‐terminal domain superfamily (CL0603.2)	oxidoreductase activity (GO:0016491), oxidoreductase activity, acting on the CH‐NH2 group of donors, NAD or NADP as acceptor (GO:0016639)
P60280 (DNA‐directed RNA polymerase subunit beta)	*RpoB*	−1,2	/	LEF‐8 like region of RNA polymerase Rpb2 (CL0410.4)	DNA‐directed 5′‐3′ RNA polymerase activity (GO:0003899), DNA binding (GO:0003677)
Q6NFB1 (ATP‐dependent Clp protease ATP‐binding subunit)	*ClpC*	−1,4	A0A854NP94 (NDP‐hexose 4‐ketoreductase)	P‐loop containing nucleoside triphosphate hydrolase superfamily (CL0023.34), AAA+ ATPase lid domain superfamily (CL0671.1)	ATP hydrolysis activity (GO:0016887), ATP binding (GO:0005524), protein binding (GO:0005515)
Q6NJF6 (DNA‐directed RNA polymerase subunit beta)	*RpoC*	−1,4	/	/	DNA‐directed 5′‐3′ RNA polymerase activity (GO:0003899), DNA binding (GO:0003677)
Q6NHH1 (Phenylalanine‐tRNA ligase beta subunit)	*PheT*	−1,7	A0A854NJU2 (Phenylalanine–tRNA ligase beta subunit)	Phenylalanine‐ and lysidine‐tRNA synthetase domain superfamily (CL0383.4), Helix‐turn‐helix clan (CL0123.18), OB fold (CL0021.18), Class II aminoacyl‐tRNA and Biotin synthetases (CL0040.17)	RNA binding (GO:0003723), phenylalanine‐tRNA ligase activity (GO:0004826), magnesium ion binding (GO:0000287), tRNA binding (GO:0000049)
P62213 (Protein RecA)	*RecA*	−2,1	A0A854NHW2 (Protein RecA (Recombinase A))	P‐loop containing nucleoside triphosphate hydrolase superfamily (CL0023.34)	ATP hydrolysis activity (GO:0016887), single‐stranded DNA binding (GO:0003697), DNA binding (GO:0003677)
Q6NF51 (ABC transport system, ATP‐binding protein)	*DIP2050*	−5,2	A0A854NJC7 (ABC transporter)	P‐loop containing nucleoside triphosphate hydrolase superfamily (CL0023.34)	ATP hydrolysis activity (GO:0016887), ATP binding (GO:0005524)
Q6NGM5 (uroporphyrinogen‐III C‐methyltransferase)	*DIP1484*	−5,5	/	/	Methyltransferase activity (GO:0008168)
Q6NGC4 (UDP‐*N*‐acetylmuramoyl‐tripeptide–d‐alanyl‐d‐alanine ligase)	*MurF*	−5,6	A0A854NCW6 (UDP‐*N*‐acetylmuramoyl‐tripeptide–d‐alanyl‐d‐alanine ligase)	MurF and HprK N‐domain‐like superfamily (CL0365.4), Mur ligase C‐terminal domain superfamily (CL0794.1), P‐loop containing nucleoside triphosphate hydrolase superfamily (CL0023.34)	ATP binding (GO:0005524), acid‐amino acid ligase activity (GO:0016881)
Q6NI09 (1D‐myo‐inositol 2‐acetamido‐2‐deoxy‐alpha‐d‐glucopyranoside deacetylase)	*MshB*	−5,9	A0A854NGL9 (1D‐myo‐inositol 2‐acetamido‐2‐deoxy‐alpha‐d‐glucopyranoside deacetylase)	/	*N*‐Acetylglucosaminylinositol deacetylase activity (GO:0035595)
Q6NIE2 (ATP‐dependent DNA helicase)	*PcrA*	−5,9	A0A854NI98 (ATP‐dependent DNA helicase)	Src homology‐3 domain (CL0010.21), P‐loop containing nucleoside triphosphate hydrolase superfamily (CL0023.34)	DNA helicase activity (GO:0003678), ATP binding (GO:0005524), DNA binding (GO:0003677)
Q6NHD5 (Protein translocase subunit SecA 2)	*SecA2*	−6,3	/	P‐loop containing nucleoside triphosphate hydrolase superfamily (CL0023.34)	ATP binding (GO:0005524), protein targeting (GO:0006605), protein import (GO:0017038)
Q6NHX6 (EcsC family protein)	*DIP1004*	−6,3	A0A854NGQ1 (EcsC family protein)	/	/
Q6NEG0 (site‐specific DNA‐methyltransferase)	*DIP2314*	−6,3	A0A854NKV2 (site‐specific DNA‐methyltransferase)	MmeI helical domain superfamily (CL0884.1), FAD/NAD(P)‐binding Rossmann fold Superfamily (CL0063.25)	DNA binding (GO:0003677)
Q6NGV3 (1‐deoxy‐d‐xylulose‐5‐phosphate synthase)	*Dxs*	−6,3	A0A854NHE6 (1‐deoxy‐d‐xylulose‐5‐phosphate synthase)	Thiamin diphosphate‐binding superfamily (CL0254.9), Transketolase C‐terminal domain‐like superfamily (CL0591.2)	1‐Deoxy‐d‐xylulose‐5‐phosphate synthase activity (GO:0008661)
Q6NHQ4 (DNA ligase)	*LigA*	−6,4	A0A854NGX2 (DNA ligase)	BRCT like (CL0459.3), ATP‐grasp superfamily (CL0179.14), OB fold (CL0021.18), Helix‐hairpin‐helix superfamily (CL0198.16)	DNA ligase (NAD+) activity (GO:0003911)
Q6NEM2 (Membrane protein)	*DIP2250*	−6,4	A0A854NF55 (LytR family transcriptional regulator)	/	/
Q6NFU7 (ATP‐dependent Clp protease ATP‐binding subunit ClpX)	*ClpX*	−6,5	A0A854NCN8 (ATP‐dependent Clp protease ATP‐binding subunit ClpX)	P‐loop containing nucleoside triphosphate hydrolase superfamily (CL0023.34), AAA+ ATPase lid domain superfamily (CL0671.1), Zinc beta‐ribbon (CL0167.15)	ATP hydrolysis activity (GO:0016887), ATP binding (GO:0005524), zinc ion binding (GO:0008270)
Q6NI10 (Secreted protein)	*DIP0970*	−6,6	A0A854NJ90 (Solute‐binding protein family 5 domain‐containing protein)	Periplasmic binding protein clan (CL0177.16)	/
Q6NI67 (Transcription‐repair‐coupling factor)	*Mfd*	−6,9	A0A854NFW3 (Transcription‐repair‐coupling factor)	Src homology‐3 domain (CL0010.21), P‐loop containing nucleoside triphosphate hydrolase superfamily (CL0023.34), UB2H/UvrB interaction domain superfamily (CL0664.1)	nucleic acid binding (GO:0003676), damaged DNA binding (GO:0003684), DNA repair (GO:0006281)
Q6NKB1 (ROK family protein)	*DIP0123*	−7	A0A854NIJ5 (ROK family protein)	Actin‐like ATPase superfamily (CL0108.16)	/
Q6NGE6 (DNA polymerase III subunit alpha)	*DnaE*	−7,3	A0A854NFC5 (DNA polymerase III subunit alpha)	Nucleotidyltransferase superfamily (CL0260.8), Helix‐hairpin‐helix superfamily (CL0198.16), Common phosphate binding‐site TIM barrel superfamily (CL0036.24), OB fold (CL0021.18)	3′‐5′ exonuclease activity (GO:0008408), nucleic acid binding (GO:0003676), catalytic activity (GO:0003824)
Q6NG58 (SPOR domain‐containing protein)	*DIP1673*	De novo translation	A0A854NBT0 (SPOR domain‐containing protein)	/	/

*Note*: Protein names of unreviewed proteins are based on ProtNLM transformer model. Biovar *mitis* homologs are based on BLASTP searches. PFAM clans and InterPro‐to‐GO annotations are based on UniProt‐annotated, predicted protein domains. LFC: Log2 fold change. De novo: Protein with non‐zero abundance exclusively detected 24 h after internalization in at least two biological replicates.

Functional characterization by means of UniProt‐annotated PFAM domains revealed that all five higher‐abundant proteins are predicted to possess enzymatic activities, three of which (TrpB, DIP0762, and Gdh) use FAD or NAD(P) as a coenzyme. The upregulated, *uncharacterized* protein DIP1797 contains a thioredoxin‐like domain and shares a strong homology with the bv. *mitis* entry A0A854NE82, which is predicted to correspond to the disulfide‐bond‐formation protein DsbA. Several lower‐abundant proteins seem to belong to P‐loop‐containing nucleoside‐triphosphate hydrolase superfamily and are predicted to possess ATP‐ and DNA‐binding molecular functions (Table 2).

Nevertheless, the reasons for the increased amounts of tryptophan synthase beta chain and the three uncharacterized proteins DIP0762, DIP1081, and DIP1797 are unclear. The increased abundance of glutamate dehydrogenase may be explained by a function in oxidative stress response as published recently for *Salmonella* [52]. Furthermore, it was shown that glutamate dehydrogenase is required to sustain *Mycobacterium bovis* BCG during infection of both murine RAW 264.7 and bone‐marrow derived macrophages [53].

**FIGURE 4:**
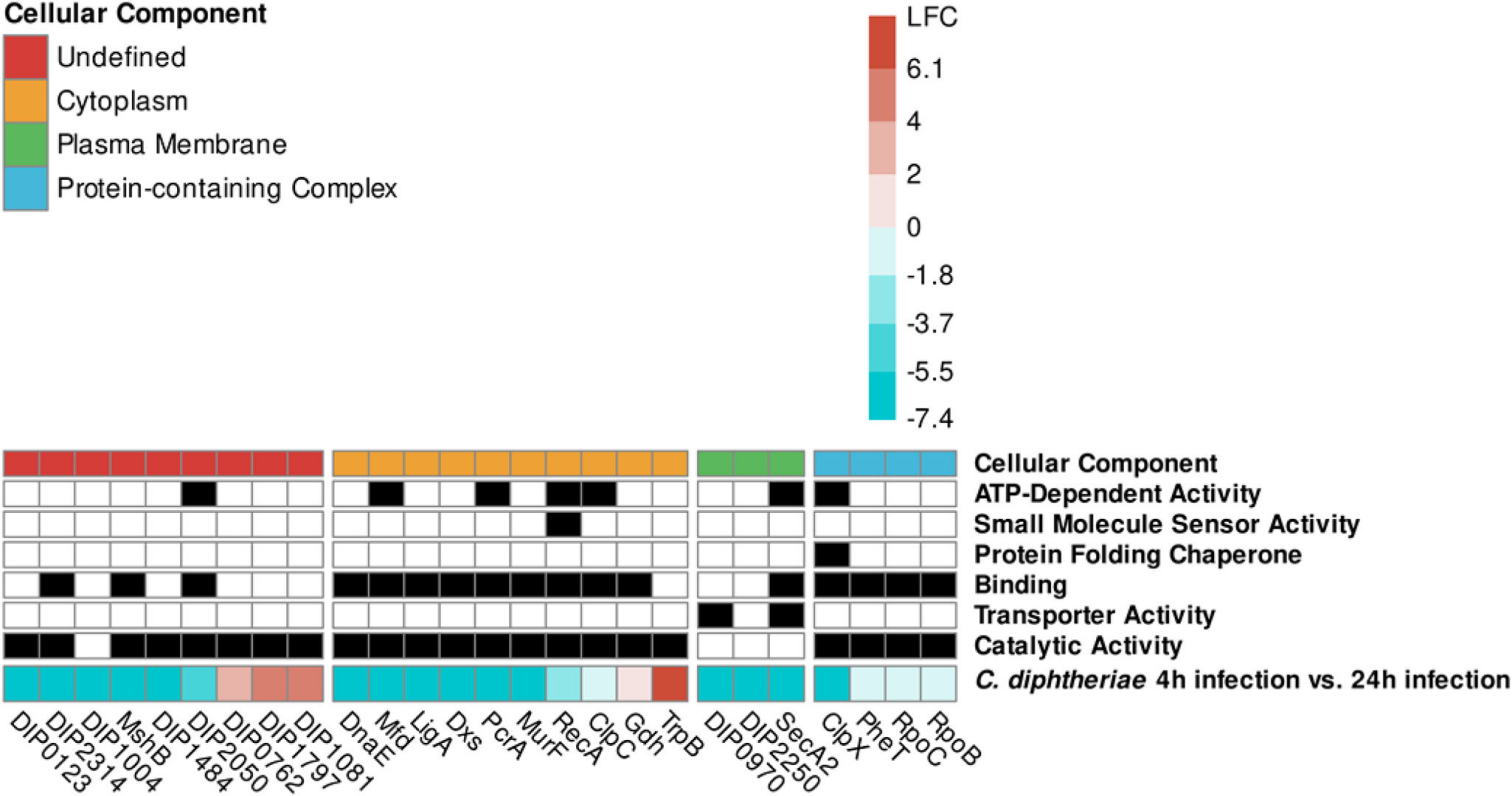
Functional characteristics of 26 significantly differentially abundant *C. diphtheriae* proteins. The proteins are sorted column‐wise by UniProt‐annotated cellular component (Gene Ontology) and log2 fold change (LFC). For each protein, UniProt‐annotated molecular function (Gene Ontology) is also shown clustered and arranged into six main categories for clarity, whose assignment is depicted by black rectangles.

From the 21 proteins with lower abundance after 24 h of internalization, two proteins are currently uncharacterized, while others are known to be involved in DNA replication (PcrA, DIP2314, LigA, DnaE), transcription (RpoB, RpoC, Mfd), protein degradation (ClpC, ClpX), and transport (DIP2050, SecA2). Interestingly, the downregulation of MshB may hint at a reduced presence of mycothiol, which would lead to a lower resistance to reactive oxygen species by *C. diphtheriae* ISS3319 24 h after internalization [54]. Cellular localization and predicted function of the proteins are shown in Figure 4.

**FIGURE 5:**
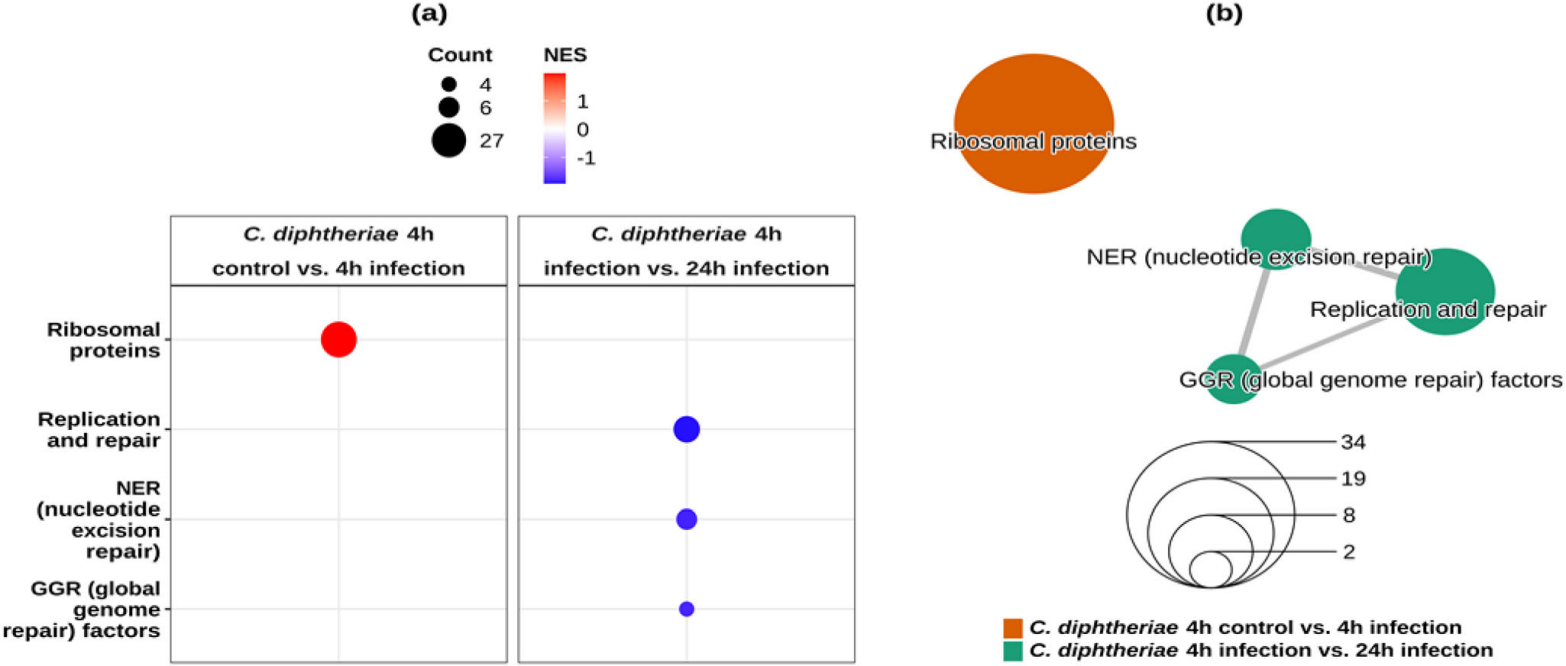
Gene set enrichment analysis (GSEA) of *C. diphtheriae* protein abundance in control and internalization conditions. (a) Dot plot of significantly enriched KEGG BRITE hierarchies. Dot size reflects the number of proteins belonging to the enrichment core, that is, proteins whose coordinated fold‐change led to the significant enrichment score. Color depicts the normalized enrichment score (NES) value of the GSEA. (b) Enrichment map of the significantly enriched BRITE hierarchies. Node size reflects the number of proteins belonging to the respective enrichment cores. If the Jaccard similarity between core enrichments of two distinct gene sets is higher than 0.2, an edge is drawn to represent functional overlaps of GSEA‐driving proteins.

In addition to the analysis of the differential abundance of single proteins in response to internalization, a GSEA was performed (Figure 5). Enriched gene sets, which here are KEGG‐derived BRITE hierarchies, were completely unconnected and oppositely regulated in the two comparisons of interest. Ribosomal proteins formed the only enriched BRITE hierarchy when comparing control and internalized bacteria after 4 h. Prolonged internalization led to a decrease of replication and repair pathways (4 vs. 24 h infection), indicating a reduced capacity of DNA maintenance and repair.

Interestingly, only one *C. diphtheriae* protein, DIP1673, was exclusively detected after 24 h of internalization, suggesting a putative important function in survival in macrophages. DIP1673 and its bv. *mitis* homolog have been annotated as “SPOR domain‐containing” proteins by the transformer model ProtNLM [31, 55]. However, no PFAM nor InterPro protein domain was assigned to either UniProt entries.

**FIGURE 6:**
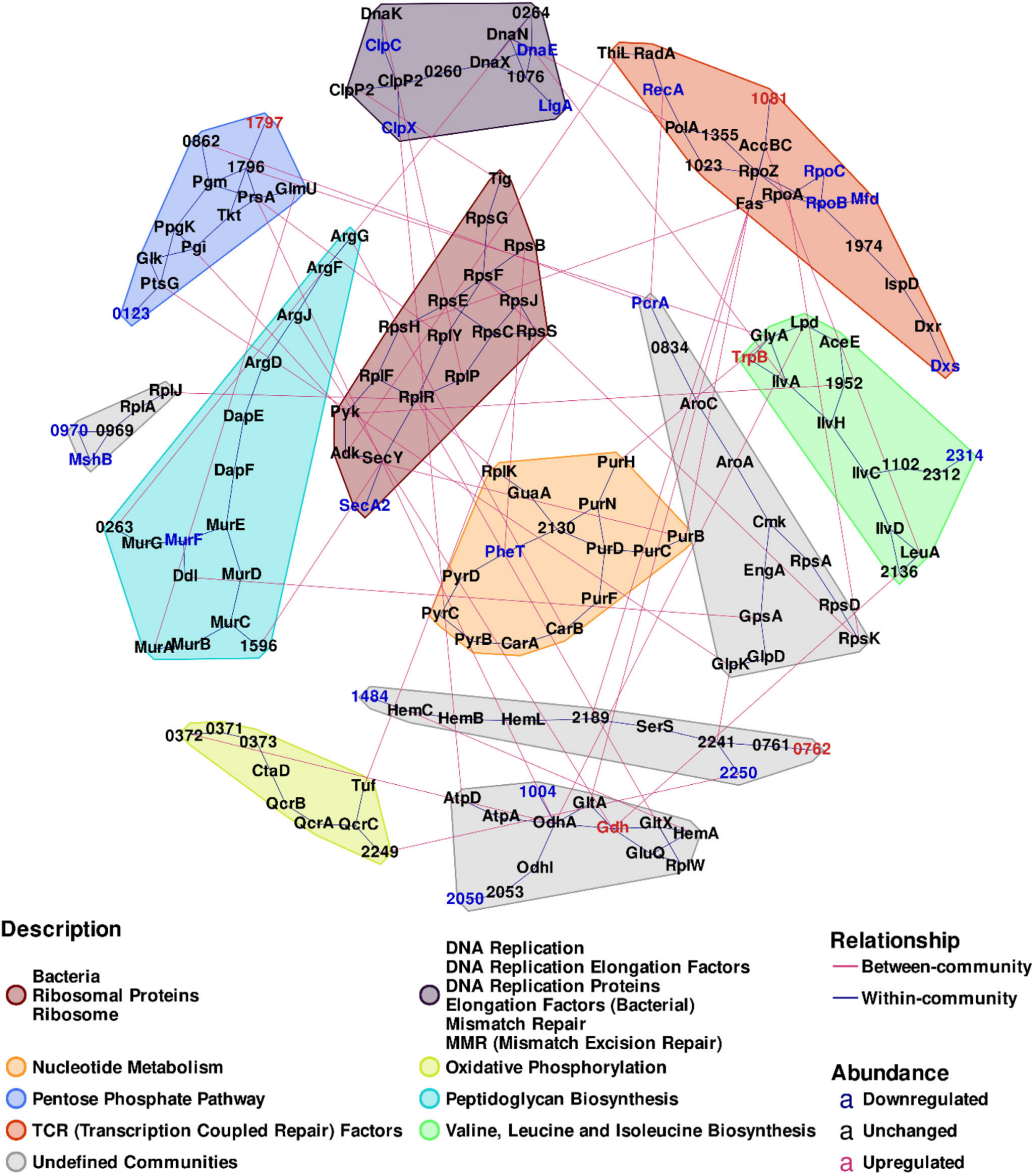
Protein interaction network based on all‐pairs‐shortest‐paths (APSPs) between differentially abundant proteins. Weighted APSPs were computed using STRING v12 functional relationships restricted to *neighborhood*, *fusion*, *coexpression*, *experimental*, and *database* (confidence cutoff: 0.4). Community descriptions were assigned via Fisher's exact test by means of 2 × 2 contingency tables between communities and KEGG's BRITE hierarchies (false discovery rate [FDR]: 5%). Only BRITE hierarchies with the highest overlap to the communities and highest odds ratios are described. “Undefined” communities did not show any significant overlap to annotated hierarchies. To shorten labels, “DIP” prefixes were removed from ordered‐locus protein names.

To further contextualize the differentially abundant proteins in *C. diphtheriae*, a STRING‐based, PPI network was produced by computing APSPs between the 27 proteins, DIP1673 included, and using only statistically‐evaluated proteins (1136). A relatively sparse network of 153 nodes and 207 edges was obtained (Figure 6). By means of Leiden clustering and Fisher's exact test, it was possible to associate some network communities to known KEGG BRITE hierarchies. Direct functional associations between the RpoB, RpoC, and MfD and between DIP0970 and MshD were found, the former within the “Transcription Coupled Repair Factors” community and the latter within an undefined containing also ribosomal proteins. Several oppositely regulated proteins cluster together, such as Gdh and DIP2050, and DIP0762 and DIP2250, however, the communities they respectively belong to do not significantly overlap with any BRITE hierarchy. Several proteins in the network play a role in the bacterial such as those in the community overlapping with the pentose phosphate pathway. The shortest paths originating from MurF and TrpB comprise proteins from the *ilv* and *arg* gene clusters, respectively, involved in isoleucine and valine, and arginine biosynthesis; the latter may also have an impact on glutamate metabolism [56, 57, 58, 59].

Unfortunately, the 24 h infection‐restricted protein DIP1673 did not show robust functional associations to any of the statistically evaluated proteins and was also not characterized in previous studies, leaving its physiological role unclear.

### 3.3 Response of Macrophages to Internalization of *C. diphtheriae* ISS3319

**FIGURE 7:**
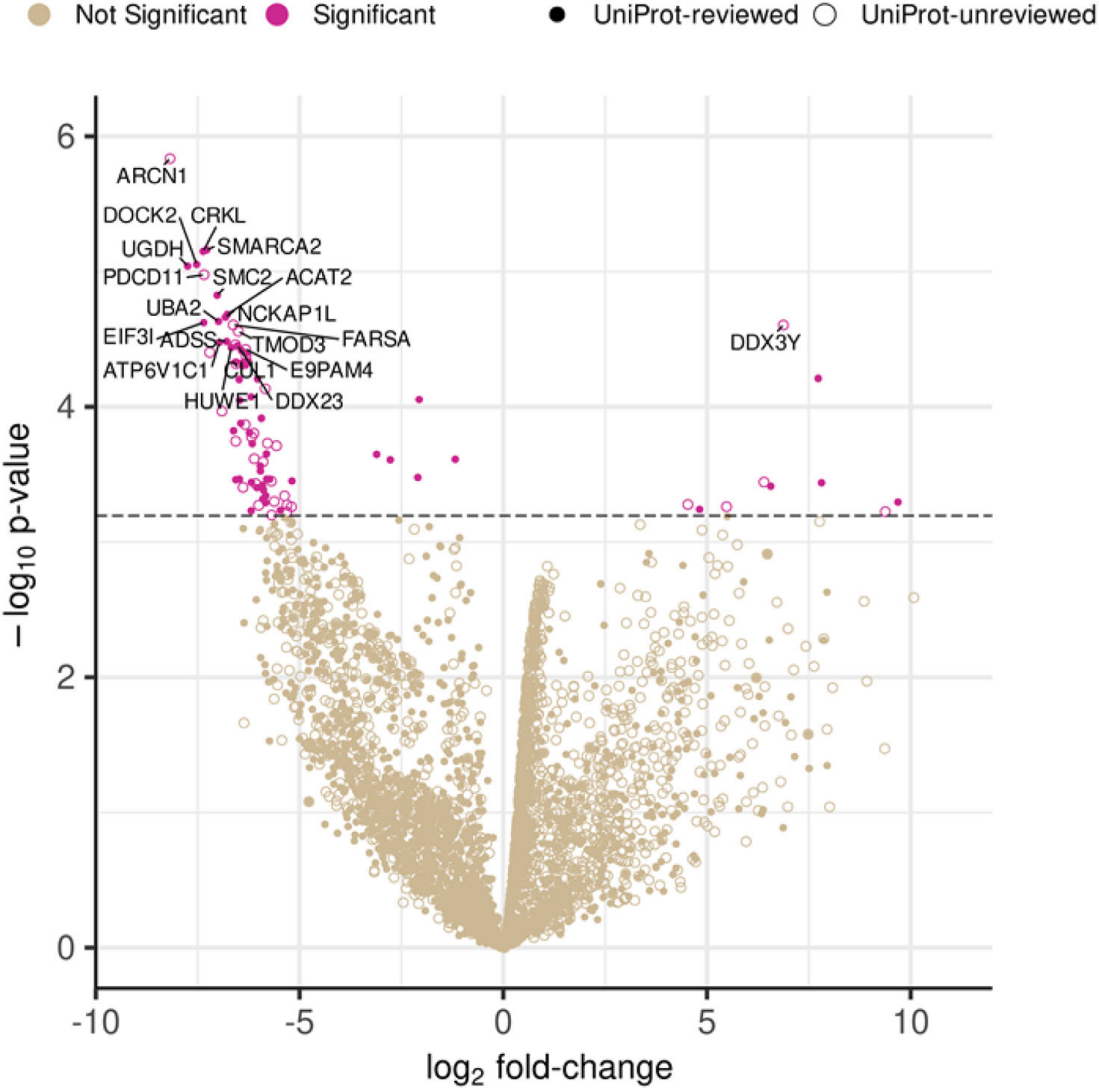
Differential protein abundance in macrophage between 4 and 24 h after internalization of *C. diphtheriae*. Log‐transformed *p* values and logarithmic fold‐changes of 8195 *H. sapiens* proteins (4093 genes) are shown. For explanations, see Figure 2.

In this study, a total of 10,882 unique macrophage proteins were detected, 8195 of which were suitable for performing differential abundance analysis, out of the 83,385 UniProt entries annotated under the reference proteome of *H. sapiens* (UniProt Proteome ID UP000005640). Although the 4 h control and infection conditions show the same number of detected proteins, fewer proteins were detected in the 24 h infection group (Table 1). Despite the fact that *C. diphtheriae* strain ISS3319 is nontoxigenic and protein synthesis of the macrophages is not impaired by the highly potent diphtheria exotoxin, in the first 4 h of *C. diphtheriae* internalization, no macrophage protein with higher or lower abundance was observed. This situation changed after 24 h of phagocytosis. Compared to the 4 h time point of *C. diphtheriae* internalization, 10 proteins (encoded by 10 genes) with increased abundance and 94 proteins (encoded by 80 genes) with diminished abundance from a total of 8195 statistically analyzed proteins (encoded by 4039 genes) were identified (Figure 7, Table 3).

**TABLE 3:** *H. sapiens* differentially abundant proteins 4 versus 24 h after phagocytosis of *C. diphtheriae*.

Uniprot ID	Protein	Description	LFC
P30049	ATP5F1D	ATP synthase subunit delta, mitochondrial OS = *Homo sapiens* OX = 9606 GN = ATP5F1D PE = 1 SV = 2	9.7
P02748	C9	Complement component C9 OS = *Homo sapiens* OX = 9606 GN = C9 PE = 1 SV = 2	7.8
P13498	CYBA	Cytochrome b‐245 light chain OS = *Homo sapiens* OX = 9606 GN = CYBA PE = 1 SV = 3	7.7
P48960	CD97	CD97 antigen OS = *Homo sapiens* OX = 9606 GN = CD97 PE = 1 SV = 4	6.6
O75915	ARL6IP5	PRA1 family protein 3 OS = *Homo sapiens* OX = 9606 GN = ARL6IP5 PE = 1 SV = 1	4.8
P09874	PARP1	Poly [ADP‐ribose] polymerase 1 OS = *Homo sapiens* OX = 9606 GN = PARP1 PE = 1 SV = 4	−1.2
P07814	EPRS	Bifunctional glutamate/proline–tRNA ligase OS = *Homo sapiens* OX = 9606 GN = EPRS PE = 1 SV = 5	−2.1
P34932	HSPA4	Heat shock 70 kDa protein 4 OS = *Homo sapiens* OX = 9606 GN = HSPA4 PE = 1 SV = 4	−2.1
Q6P2Q9	PRPF8	Pre‐mRNA‐processing‐splicing Factor 8 OS = *Homo sapiens* OX = 9606 GN = PRPF8 PE = 1 SV = 2	−2.8
Q14204	DYNC1H1	Cytoplasmic dynein 1 heavy chain 1 OS = *Homo sapiens* OX = 9606 GN = DYNC1H1 PE = 1 SV = 5	−3.1
Q9UBU9	NXF1	Nuclear RNA export Factor 1 OS = *Homo sapiens* OX = 9606 GN = NXF1 PE = 1 SV = 1	−5.2
Q9Y6W5	WASF2	Wiskott‐Aldrich syndrome protein family member 2 OS = *Homo sapiens* OX = 9606 GN = WASF2 PE = 1 SV = 3	−5.5
Q96ST3	SIN3A	Paired amphipathic helix protein Sin3a OS = *Homo sapiens* OX = 9606 GN = SIN3A PE = 1 SV = 2	−5.7
A0AVT1	UBA6	Ubiquitin‐like modifier‐activating enzyme 6 OS = *Homo sapiens* OX = 9606 GN = UBA6 PE = 1 SV = 1	−5.8
P11216	PYGB	Glycogen phosphorylase, brain form OS = *Homo sapiens* OX = 9606 GN = PYGB PE = 1 SV = 5	−5.8
Q09161	NCBP1	Nuclear cap‐binding protein subunit 1 OS = *Homo sapiens* OX = 9606 GN = NCBP1 PE = 1 SV = 1	−5.8
Q13045	FLII	Protein flightless‐1 homolog OS = *Homo sapiens* OX = 9606 GN = FLII PE = 1 SV = 2	−5.8
A6NHR9	SMCHD1	Structural maintenance of chromosomes flexible hinge domain‐containing protein 1 OS = *Homo sapiens* OX = 9606 GN = SMCHD1 PE = 1 SV = 2	−5.9
P26358	DNMT1	DNA (cytosine‐5)‐methyltransferase 1 OS = *Homo sapiens* OX = 9606 GN = DNMT1 PE = 1 SV = 2	−5.9
Q9GZL7	WDR12	Ribosome biogenesis protein WDR12 OS = *Homo sapiens* OX = 9606 GN = WDR12 PE = 1 SV = 2	−5.9
Q68CZ2	TNS3	Tensin‐3 OS = *Homo sapiens* OX = 9606 GN = TNS3 PE = 1 SV = 2	−5.9
O75436	VPS26A	Vacuolar protein sorting‐associated protein 26A OS = *Homo sapiens* OX = 9606 GN = VPS26A PE = 1 SV = 2	−6.0
Q2TAY7	SMU1	WD40 repeat‐containing protein SMU1 OS = *Homo sapiens* OX = 9606 GN = SMU1 PE = 1 SV = 2	−6.0
Q9BVJ6	UTP14A	U3 small nucleolar RNA‐associated protein 14 homolog A OS = *Homo sapiens* OX = 9606 GN = UTP14A PE = 1 SV = 1	−6.0
Q9Y5×1	SNX9	Sorting nexin‐9 OS = *Homo sapiens* OX = 9606 GN = SNX9 PE = 1 SV = 1	−6.0
Q9NRL2	BAZ1A	Bromodomain adjacent to zinc finger domain protein 1A OS = *Homo sapiens* OX = 9606 GN = BAZ1A PE = 1 SV = 2	−6.2
Q9UI12	ATP6V1H	V‐type proton ATPase subunit H OS = *Homo sapiens* OX = 9606 GN = ATP6V1H PE = 1 SV = 1	−6.2
Q2VIR3	EIF2S3B	Eukaryotic translation initiation Factor 2 subunit 3B OS = *Homo sapiens* OX = 9606 GN = EIF2S3B PE = 2 SV = 2	−6.2
Q9Y5×3	SNX5	Sorting nexin‐5 OS = *Homo sapiens* OX = 9606 GN = SNX5 PE = 1 SV = 1	−6.2
P49756	RBM25	RNA‐binding protein 25 OS = *Homo sapiens* OX = 9606 GN = RBM25 PE = 1 SV = 3	−6.3
P52948	NUP98	Nuclear pore complex protein Nup98‐Nup96 OS = *Homo sapiens* OX = 9606 GN = NUP98 PE = 1 SV = 4	−6.3
Q9NZB2	FAM120A	Constitutive coactivator of PPAR‐gamma‐like protein 1 OS = *Homo sapiens* OX = 9606 GN = FAM120A PE = 1 SV = 2	−6.3
O00186	STXBP3	Syntaxin‐binding protein 3 OS = *Homo sapiens* OX = 9606 GN = STXBP3 PE = 1 SV = 2	−6.4
P61081	UBE2M	NEDD8‐conjugating enzyme Ubc12 OS = *Homo sapiens* OX = 9606 GN = UBE2 M PE = 1 SV = 1	−6.4
Q16719	KYNU	Kynureninase OS = *Homo sapiens* OX = 9606 GN = KYNU PE = 1 SV = 1	−6.4
Q9BUQ8	DDX23	Probable ATP‐dependent RNA helicase DDX23 OS = *Homo sapiens* OX = 9606 GN = DDX23 PE = 1 SV = 3	−6.4
O76094	SRP72	Signal recognition particle subunit SRP72 OS = *Homo sapiens* OX = 9606 GN = SRP72 PE = 1 SV = 3	−6.5
P20073	ANXA7	Annexin A7 OS = *Homo sapiens* OX = 9606 GN = ANXA7 PE = 1 SV = 3	−6.5
Q02790	FKBP4	Peptidyl‐prolyl cis‐trans isomerase FKBP4 OS = *Homo sapiens* OX = 9606 GN = FKBP4 PE = 1 SV = 3	−6.5
Q13616	CUL1	Cullin‐1 OS = *Homo sapiens* OX = 9606 GN = CUL1 PE = 1 SV = 2	−6.5
P51610	HCFC1	Host cell Factor 1 OS = *Homo sapiens* OX = 9606 GN = HCFC1 PE = 1 SV = 2	−6.6
Q7Z417	NUFIP2	Nuclear fragile X mental retardation‐interacting protein 2 OS = *Homo sapiens* OX = 9606 GN = NUFIP2 PE = 1 SV = 1	−6.6
Q8TDZ2	MICAL1	[F‐actin]‐monooxygenase MICAL1 OS = *Homo sapiens* OX = 9606 GN = MICAL1 PE = 1 SV = 2	−6.6
Q7Z6Z7	HUWE1	E3 ubiquitin‐protein ligase HUWE1 OS = *Homo sapiens* OX = 9606 GN = HUWE1 PE = 1 SV = 3	−6.7
P30520	ADSS	Adenylosuccinate synthetase isozyme 2 OS = *Homo sapiens* OX = 9606 GN = ADSS PE = 1 SV = 3	−6.8
P55160	NCKAP1L	Nck‐associated protein 1‐like OS = *Homo sapiens* OX = 9606 GN = NCKAP1L PE = 1 SV = 3	−6.8
Q9BWD1	ACAT2	Acetyl‐CoA acetyltransferase, cytosolic OS = *Homo sapiens* OX = 9606 GN = ACAT2 PE = 1 SV = 2	−6.8
O95347	SMC2	Structural maintenance of chromosomes protein 2 OS = *Homo sapiens* OX = 9606 GN = SMC2 PE = 1 SV = 2	−7.0
P21283	ATP6V1C1	V‐type proton ATPase subunit C 1 OS = *Homo sapiens* OX = 9606 GN = ATP6V1C1 PE = 1 SV = 4	−7.0
Q9UBT2	UBA2	SUMO‐activating enzyme subunit 2 OS = *Homo sapiens* OX = 9606 GN = UBA2 PE = 1 SV = 2	−7.0
P51531	SMARCA2	Probable global transcription activator SNF2L2 OS = *Homo sapiens* OX = 9606 GN = SMARCA2 PE = 1 SV = 2	−7.3
P46109	CRKL	Crk‐like protein OS = *Homo sapiens* OX = 9606 GN = CRKL PE = 1 SV = 1	−7.4
Q13347	EIF3I	Eukaryotic translation initiation Factor 3 subunit I OS = *Homo sapiens* OX = 9606 GN = EIF3I PE = 1 SV = 1	−7.4
Q92608	DOCK2	Dedicator of cytokinesis protein 2 OS = *Homo sapiens* OX = 9606 GN = DOCK2 PE = 1 SV = 2	−7.5
O60701	UGDH	UDP‐glucose 6‐dehydrogenase OS = *Homo sapiens* OX = 9606 GN = UGDH PE = 1 SV = 1	−7.7

*Note*: Only Uniprot‐reviewed entries are shown, see Supporting Information for the complete list at https://zenodo.org/doi/zenodo.12706139. LFC: Log2 fold change.

**FIGURE 8:**
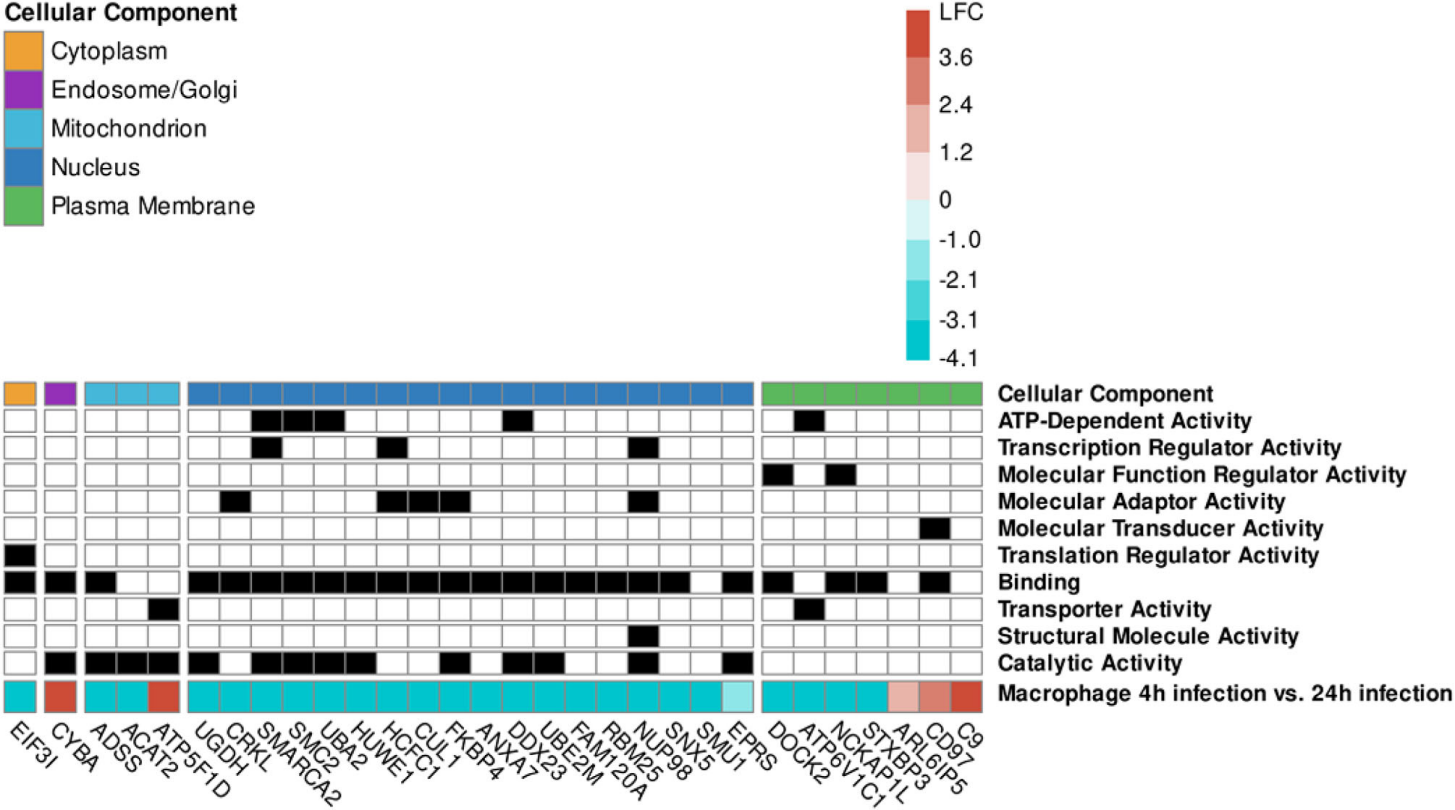
Functional characteristics of differentially expressed *H. sapiens*, UniProt‐reviewed proteins. The 5 proteins with positive log2 fold changes (LFCs) and the 25 significantly less abundant proteins with the smallest *p* values are depicted. The proteins are sorted column‐wise by UniProt‐annotated cellular component (Gene Ontology) and LFC. For explanations, see Figure 4.

Among the proteins with increased abundance, proteins relevant for the innate immune response such as the complement system factor C9 and the cytokine CD97 were found. The membrane‐localized protein ARLP6IP5 was also upregulated, and its mouse homolog was shown to be relevant for negatively regulating glutamate uptake, a relevant metabolite in host–pathogen interactions [60, 61]. Remarkably, the regulatory subunit H of the V‐type proton ATPase (ATP6V1H, Table 3), which is required for ATP catalysis but not the assembly of V‐ATPase, and the C1 subunit (ATP6V1C1), which is part of the catalytic V1 domain, were both downregulated 24 h after infection. This may indicate a reduction in active acidification of the phagosome. In Figure 8, the GO annotation of 30 out of 56 UniProt‐reviewed proteins is shown. This set was constructed by including the 5 significantly more abundant proteins and 25 significantly less abundant ones with the smallest *p* values, with respect to the 24 h versus 4 h comparison. Notably, the most significantly less abundant proteins had a nuclear localization. Three proteins with transcription regulator activity were significantly less abundant 24 h after infection compared to 4 h (Figure 8). HCFC1 and SIN3A are known to interact in the context of gene transcription in immune response [62], while SMARCA2 has been characterized in cell differentiation and cancer, and it may regulate the expression of interferon‐dependent genes [63, 64].

**FIGURE 9:**
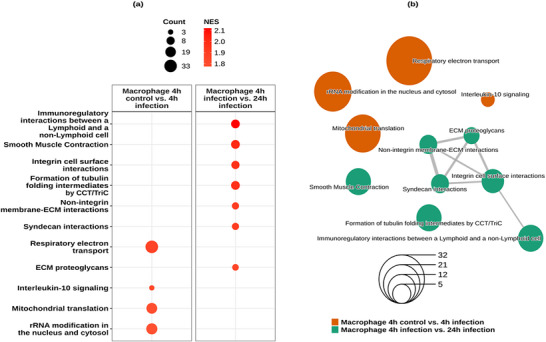
Gene set enrichment analysis (GSEA) of macrophage protein expression in control and infection conditions. (a) Dot plot of significantly enriched Reactome pathways. Dot size refers to the number of proteins belonging to the enrichment core, that is, proteins whose coordinated fold‐change led to the significant enrichment score. Color depicts the normalized enrichment score (NES) value of the GSEA. (b) Enrichment map of the significantly enriched Reactome pathways. For explanations, see Figure 5.

Next, GSEA was performed on macrophage proteins, similarly to what shown for *C. diphtheriae* (Figure 9). Enriched gene sets, which here are Reactome pathways, were completely unconnected but consistently upregulated in the two comparisons of interest. Interleukin 10 signaling may hint at the immune‐regulatory cascade that can lead to antigen load into MHC‐I complexes and production of lymphocyte‐recruiting cytokines 24 h after infection (see CD97). Mitochondrial respiration and therefore active energy production 4 h after infection is implied by the pathways “respiratory electron transport” and “mitochondrial translation”. Twenty‐four hours after infection, a coordinated protein expression that could trigger an adaptive immune system response was observed (“Immunoregulatory interactions between…”). This is reflected by positive log fold‐changes observed for MHC‐I proteins like HLA‐A, B, and C, albeit differential protein abundance could not address which exact loci were significantly upregulated. Interestingly, a remodeling of integrin‐ and nonintegrin‐mediated interaction within the extracellular matrix was also observed 24 h after infection. “Smooth muscle contraction” and “Formation of tubulin…” hint at activation of the cytoskeleton (tropomyosin/tropomodulin and tubulin‐associated proteins).

Finally, 17 macrophage proteins were first detected only 24 h after infection (Table 4). Several of these are UniProt‐unreviewed. Still, the UniProt‐reviewed proteins translated from CCL5 and EBI3 (IL‐27 beta subunit) loci further hint at the onset of an immune response aimed at regulating and activating B and T cells. It is worth noting that IL‐27 has been described as an antiinflammatory cytokine and that internalized EBI3 has been shown to have antiapoptotic activity in murine macrophages infected with *M. tuberculosis* [65, 66].

**TABLE 4:** De novo protein synthesis in macrophages 24 h after phagocytosis of *C. diphtheriae*.

UniProt ID	Protein	Description	UniProt review status
A0A087×090	FDPS	Farnesyl diphosphate synthase (farnesyl pyrophosphate synthetase, dimethylallyltranstransferase, geranyltranstransferase), isoform CRAa OS = *Homo sapiens* OX = 9606 GN = FDPS PE = 1 SV = 1	Unreviewed
A0A0G2JI36	HLA‐A	HLA Class I histocompatibility antigen, A‐3 alpha chain OS = *Homo sapiens* OX = 9606 GN = HLA‐A PE = 1 SV = 1	Unreviewed
A0A0U1RQR6	RTN4	Reticulon (fragment) OS = *Homo sapiens* OX = 9606 GN = RTN4 PE = 1 SV = 1	Unreviewed
A0A2R8YEU4	TPM4	Tropomyosin alpha‐4 chain (fragment) OS = *Homo sapiens* OX = 9606 GN = TPM4 PE = 1 SV = 1	Unreviewed
A7XZE4	TPM2	Beta tropomyosin isoform OS = *Homo sapiens* OX = 9606 GN = TPM2 PE = 1 SV = 1	Unreviewed
D6RBN5	OCIAD1	OCIA domain‐containing protein 1 OS = *Homo sapiens* OX = 9606 GN = OCIAD1 PE = 1 SV = 1	Unreviewed
F8VRK0	TUBA1B	Tubulin alpha‐1B chain (fragment) OS = *Homo sapiens* OX = 9606 GN = TUBA1B PE = 1 SV = 1	Unreviewed
F8VRZ4	TUBA1A	Tubulin alpha‐1A chain (fragment) OS = *Homo sapiens* OX = 9606 GN = TUBA1A PE = 1 SV = 1	Unreviewed
F8VVD3	OSBPL8	Oxysterol‐binding protein‐related protein 8 (fragment) OS = *Homo sapiens* OX = 9606 GN = OSBPL8 PE = 1 SV = 1	Unreviewed
F8VXH9	PCBP2	Poly(rC)‐binding protein 2 (fragment) OS = *Homo sapiens* OX = 9606 GN = PCBP2 PE = 1 SV = 1	Unreviewed
H0Y8Y7	TCOF1	Treacle protein OS = *Homo sapiens* OX = 9606 GN = TCOF1 PE = 1 SV = 2	Unreviewed
H0Y9M8	NDUFS4	NADH dehydrogenase [ubiquinone] iron‐sulfur protein 4, mitochondrial (Fragment) OS = *Homo sapiens* OX = 9606 GN = NDUFS4 PE = 1 SV = 1	Unreviewed
H0YL80	TPM1	Tropomyosin alpha‐1 chain (fragment) OS = *Homo sapiens* OX = 9606 GN = TPM1 PE = 1 SV = 1	Unreviewed
P13501	CCL5	C‐C motif chemokine 5 OS = *Homo sapiens* OX = 9606 GN = CCL5 PE = 1 SV = 3	Reviewed
P17066	HSPA6	Heat shock 70 kDa protein 6 OS = *Homo sapiens* OX = 9606 GN = HSPA6 PE = 1 SV = 2	Reviewed
Q14213	EBI3	Interleukin‐27 subunit beta OS = *Homo sapiens* OX = 9606 GN = EBI3 PE = 1 SV = 2	Reviewed
Q8WXG1	RSAD2	Radical S‐adenosyl methionine domain‐containing protein 2 OS = *Homo sapiens* OX = 9606 GN = RSAD2 PE = 1 SV = 1	Reviewed

*Note*: These proteins were detected in two biological replicates out of three in the 24 h infection group, but were not detected in any biological replicate of the other two conditions.

## 4 Discussion

Up to now, *C. diphtheriae* host–pathogen interaction studies focused on distinct proteins, which were suspected to be directly involved in the infection process (e.g., see Section 1 and [67, 68, 69]) or in pathways important to survive in the host, for example, iron acquisition and heme utilization [70, 71, 72, 73]. In the frame of this study, a global analysis was carried out without the aim to characterize a distinct protein or pathway, but to get an unbiased view on bacterial and cellular proteome adaptations from the same samples at the same time. To our best knowledge, this is the first proteome analysis of a *C. diphtheriae* macrophage infection experiment.

Astonishingly, the characterization of the *C. diphtheriae* proteome during internalization revealed only 5 proteins with higher and 21 proteins with lower abundance between 4 and 24 h of infection and 1 protein synthesized de novo after 24 h of infection. The unexpectedly low number of upregulated proteins may be the result of a preadaptation of *C. diphtheriae* to the harsh conditions of the phagosome as discussed previously. In general, corynebacteria seem to be able to cope with unfavorable environmental conditions and multiple stresses, making major proteome adaptation unnecessary [74]. Furthermore, components of cell culture medium in which the macrophages are sustained influence the bacterial proteome. As shown previously, the amount of proteins related to iron limitation and under control of DtxR as well as those involved in oxidative or nitrosative stress response were increased in cell culture medium without host cell contact, while other proteins involved in pathogenesis were already found in bacteria grown in complex medium (BHI) and in cell culture components: the multifunctional protein DIP0733, the conserved hypothetical protein DIP1546, and the resuscitation promoting factor RpfB DIP0874 [50]. To get deeper insight into the infection process, future studies may focus especially on the uncharacterized proteins DIP0762, DIP1081, DIP1673, and DIP1797 and their physiological role in host–pathogen interaction.

Proteins with lower abundance upon internalization were, as far as annotated, mainly involved in DNA replication, transcription, protein degradation, and secretion. MshB, a key enzyme in the biosynthesis of mycothiol, which acts as a cofactor in redox homeostasis maintenance, is downregulated as well. This may indicate that the increase in ROS and RNS inside the macrophage phagosome could lead to significant DNA and protein damage, such as cysteine oxidization. This could force the reduction of nonessential molecular processes (e.g., protein secretion) and reduce the biosynthesis of metabolites having amino acids and especially cysteine as precursors, like mycothiol. Nevertheless, further metabolomic studies should be carried out to elucidate *C. diphtheriae* metabolism upon infection, especially upon deprivation of important metabolites like glutamate, as hinted by ARL6IP5 upregulation in macrophages and by the network analysis of differentially abundant bacterial proteins.

The role of upregulated and de novo synthesized proteins (DIP0762, DIP1673, and DIP1797) putatively involved in pathogenicity, but with little to no functional annotation, may be experimentally addressed in future studies by gene knockout experiments, thus evaluating adhesion, survival, and cytotoxicity of respective mutant strains in comparison to the wild‐type.

On the macrophage side, most differentially expressed proteins have lower rather than higher abundances 24 h after infection, compared to 4 h, similar to *C. diphtheriae*. However, the results suggest that this might be due to a resolution of the innate immune response. For instance, the subunits of the V‐ATPase complex ATP6V1H and ATP6V1C1 are downregulated, which suggests an overall deactivation of the proton pump necessary for phagolysosome acidification during the respiratory burst. Taken together with the previously described delay of phagolysosome maturation, it may be speculated that phagolysosomes first maturate and later deactivate their bactericidal mechanisms within the 20 h that separate the two investigated time points. After that, it seems that macrophages prompt an adaptive immune response against *C. diphtheriae*, as highlighted by pathway enrichment (Figure 9) and chemokine upregulation, like CD97 and the de novo translated proteins CCL5 and EBI3. Still, further research is needed to address the kinetics of phagosome maturation and antigen presentation in the context of *C. diphtheriae* infection. The design of future studies may avoid separate cell lysis of macrophages and bacteria since this procedure may lead to an underestimation of macrophage membrane proteins and implement different time points. Furthermore, it seems to be necessary to employ additional omics and computational approaches to effectively identify host–pathogen interactions at the molecular level. Motivated by this study, we are currently working on a systems biology approach to reconstruct a database‐ and transcriptomics‐derived molecular interaction network of *C. diphtheriae* and human macrophages.

The proteome analysis of *C. diphtheriae* and macrophages presented here indicated that nontoxigenic *C. diphtheriae* are preadapted to the infection process, at least when a cell culture medium is used as control. Only a very limited number of proteins showed an in‐creased abundance upon internalization under these conditions. Many of these are un‐characterized and their physiological function needs to be elucidated in the future. Prolonged internalization led to a reduction of the general cell maintenance apparatus. Macrophages seem to react with a transient activation of their bactericidal mechanisms followed by an adaptive immune response and chemokine upregulation in response to the internalization of *C. diphtheriae*.

## Conflicts of Interest

The authors declare no conflicts of interest.

## Data Availability

All data presented here are openly available at https://zenodo.org/doi/10.5281/zenodo.12706139 and have been deposited at MassIVE (ProteomeExchange Id. PXD061792, ftp://massive.ucsd.edu/v09/MSV000097308/).
